# Seeking orderness out of the orderless movements: an up-to-date review of the biomechanics in clear aligners

**DOI:** 10.1186/s40510-024-00543-1

**Published:** 2024-11-18

**Authors:** Jiawei Li, Jiarui Si, Chaoran Xue, Hui Xu

**Affiliations:** 1 B.S, State Key Laboratory of Oral Diseases & National Clinical Research Center for Oral Diseases, West China Hospital of Stomatology, Sichuan University, Chengdu, China; 2 State Key Laboratory of Oral Diseases & National Clinical Research Center for Oral Diseases, Department of Orthodontics, West China Hospital of Stomatology, Sichuan University, Chengdu, China

**Keywords:** Clear aligner, Biomechanics, Tooth movement

## Abstract

**Introduction:**

Although with increasing popularity due to aesthetic appeal and comfort, clear aligners (CAs) are facing challenges in efficacy and predictability. Advancement in the underlying biomechanical field is crucial to addressing these challenges. This paper endeavors to provide a comprehensive framework for understanding the biomechanics of CA and enlightening biomechanics-based improvements on treatment strategies.

**Methods:**

A thorough review of the English-language literature accessible through PubMed and Google Scholar, without any publication year restrictions, was undertaken to unravel the biomechanical aspects of CA.

**Results:**

This review presented an up-to-date understanding of aligner biomechanics arranged by the framework of the material-dependent mechanical characteristics of CA, the geometric characteristics-dependent force transmission of the CA system, methods for studying the biomechanics of CA, and the biomechanical analyses for different types of tooth movement.

**Conclusions:**

Biomechanics should be the fundamental concern for concepts, methods and adjuncts attempting to enhance the accuracy and predictability of tooth movement induced by CA. Improvement on material properties and alteration of geometric design of CA are two main approaches to develop biomechanically optimized force system. Exploration of real-world force sensing and monitoring system would make substantial progresses in aligner biomechanics.

## Introduction

In 1940, Kesling first introduced the concept of “clear aligners” [1]. In 1971, inspired by Kesling, Ponitz coined the term “the invisible retainer” [2]. The demand of aesthetic appeal and comfort had driven constant upgrading and improvement of clear aligners (CAs), however, slowly during a long period of time. Over the past 20 years, the advancement of computer technologies and materials science has boosted fast progress in this process. We have witnessed a tremendous increase in the development and application of clear aligners as well as in the range of clinical scenarios they were applied in [3, 4]. CAs can be applied to a variety of dental issues with aesthetic and lightweight experiences [5], and are more appealing than fixed appliances, especially to young people [6]. The good performances of CAs in vertical control have in some cases offered treatment alternatives better suit both patients’ and orthodontists’ desires [7]. However, challenges are still faced in the cases that require tooth movements such as long-distance rotation, root torquing and translation.

Although with the growing and seemingly thriving clear aligners market, the efficacy and scope of clear aligners were hampered by limited knowledge of the biomechanical aspects of these appliances. Over the years, a significant amount of research have been dedicated solely to examining the effectiveness of tooth alignment using the clear-aligner system [8]. It was not until the past decade or so that researchers gradually began to focus on the biomechanics of clear aligners [9–11]. In 2008, Kwon et al. demonstrated the force and energy transmission characteristics of clear aligner materials [12]. In 2014, Simon et al. utilized a measurement and sensor system to measure the forces and moments for three types of tooth movements [9]. Since finite element analysis (FEA) was first applied in the field of orthodontics in 1980 [13], an increasing number of researchers have recently favored this technique as a means to simulate tooth movement and to evaluate the stresses generated within periodontal tissues, due to its non-invasive and accurate properties [14–29]. There is an intricate and complex field that needs to be explored, constituted by material properties, manufacturing precision, attachment design, virtual design of the sequence and amount of tooth movement, accuracy of aligner placement and patient compliance [30, 31].

Despite the abundance of documents on the treatment efficacy and strategy improvement [32], very few studies had focused on the biomechanical limitations [32, 33]. A comprehensive reviewing of the biomechanical aspects of clear aligners is still lacking. In this study, we provided an up-to-date review focusing on the biomechanical characteristics of clear aligners, as well as uncovering their links to the efficacy in various types of tooth movement, within the existing academic domain.

## Materials and methods

**Fig. 1:**
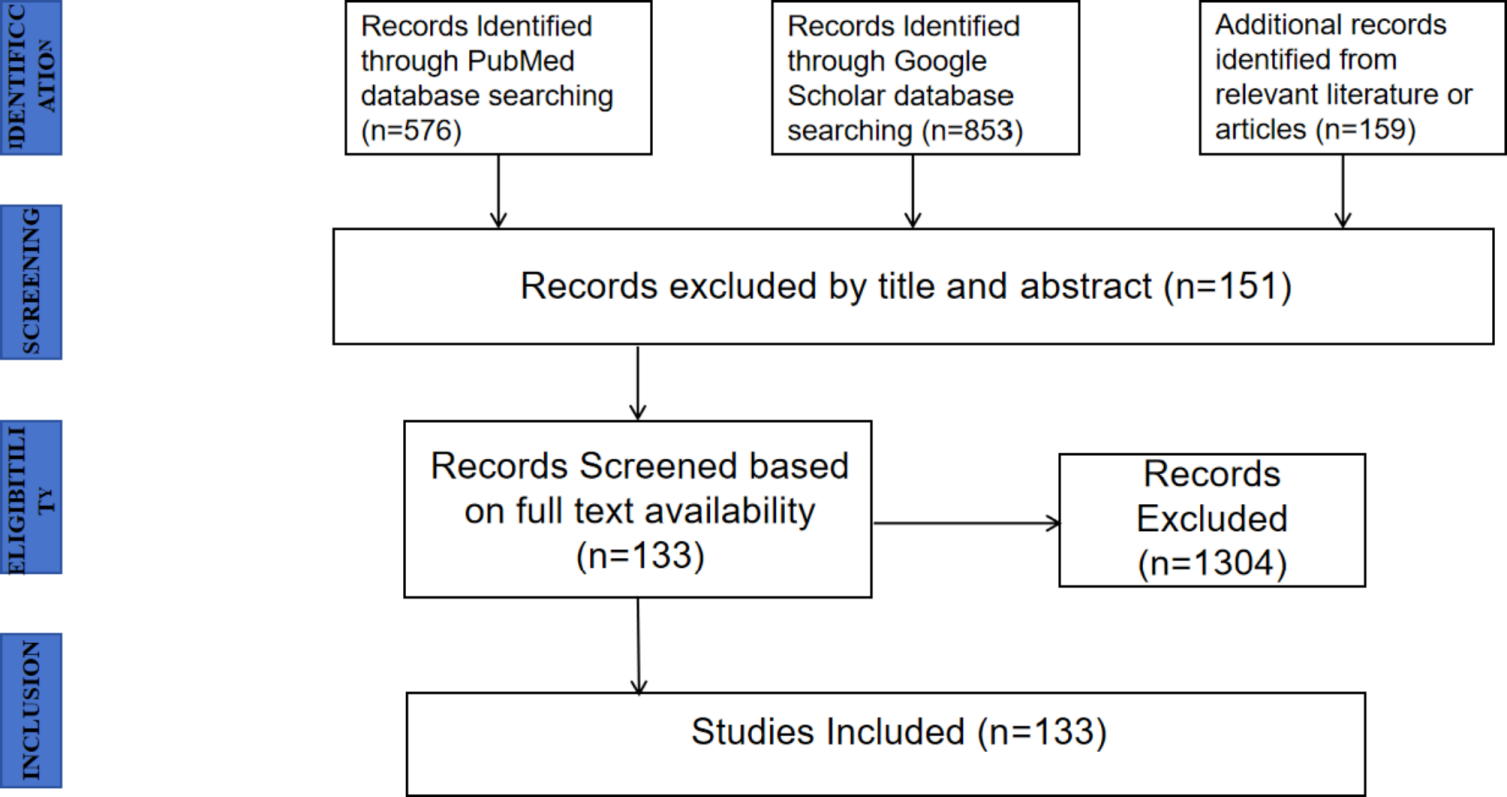
PRISMA flowchart for selection of studies for the narrative review

A non-systematic narrative review was conducted with Boolean operators using the PubMed and Google Scholar databases (Fig. 1). The following search strategy and keywords were used: “(Clear Aligners)” and “(Clear aligners) AND (biomechanics)” and “(Clear aligners) AND (force)” and “(Aligners vs. Braces)” and “(materials) AND (clear aligners)” and “(elasticity modulus) AND (aligners)” and “(stress relaxation) AND (aligners)” and “(ultimate tensile strength) AND (aligners)” and “(attachments) AND (aligners)” and “(Clear aligner) AND (tooth movements)” and “(Clear aligners) AND (finite element analysis)” and “(tooth movement) AND (aligners)”and “(rotation) AND (aligners)” and “(distalisation) AND (aligners)” and “(intrusion) AND (aligners)” and “(elasticity modulus) AND (aligners)” and “(En-masse retraction) AND (Clear aligners)” and “(torque) AND (aligners)” and “(mesialization) AND (aligners)”.

## Materials and mechanical properties of clear aligners

Tooth movement is controlled by both the applied force and the biological response of the periodontal tissues [34]. The orthodontic force is provided by the elastic polymer corrector worn on the teeth [35], leading to tipping, translation or rotation of the teeth. The combination of these basic movements forms various complex tooth movements. The force system that clear aligners provide is complicated by the lack of specific points of force application, mismatch with the anatomical structure of the teeth, incompatibility of the biomechanical properties of the dental-alveolar complex with the material properties of the aligners, as well as mismatch between the aligners and the geometric shape of the dental arch [32]. Compared to fixed appliances, clear aligners struggle to apply stable, predictable, and gentle forces to the teeth [35].

An ideal orthodontic appliance should exhibit consistent force delivery, rigidity, and high yield strength, ensuring that force is transmitted within its elastic range [32]. This means that the material must withstand a certain degree of deformation while still delivering the required force system [36]. Research has demonstrated that orthodontic tooth movement (OTM) is most effective when the periodontal membrane is subjected to an optimal hydrostatic pressure of 4.7-16KPa [37, 79]. Under identical activation conditions, the mechanical properties of the aligner influence the average strain rate, which in turn impacts the hydrostatic pressure in the periodontal membrane. Li et al. explored the optimal activation level for the distal edge of the canine in aligners with an elastic modulus of 528 MPa and a Poisson’s ratio of 0.36 [37]. Kwon et al. determined that the ideal activation range for various Essix materials is between 0.2 and 0.5 mm [12]. Evidence is still lacking on the commonly-recognized optimal parameter ranges for material characteristics. It would be beneficial to have further research conducted to uncover these parameters for specific materials.

### Evolving of the materials for CA

In 1971, Ponitz proposed the use of polymethyl methacrylate to manufacture invisible aligners. However, those aligners could only achieve limited tooth movement due to limitations of the material [2]. In 1985, McNamara modified Ponitz’s technique and made invisible retainers which enabled retaining of alignment and further refining [1]. In 1998, the material primarily used in fabrication of aligners was single-layer polyurethane [32], which was subsequently replaced by thermoplastic polyurethane (TPU), polypropylene, polycarbonate and polyethylene terephthalate (PET) [38, 39]. These materials, compared to single-layer polyurethane, provide more stable and greater elasticity [38]. The mainstream material for CA is currently polyethylene terephthalate glycol (PETG) [40]. Three dimensional (3-D) printing materials have emerged as the prevailing trend in development of future CA [41]. Various types of high-tech materials such as SmartTrack were reported to possess good mechanical properties that prevent stress relaxation over short periods and enable more predictable tooth movement [41, 42]. The mainstream CA materials can be currently divided into two categories, thermoplastic and 3-D printing materials [41].

### Comparison of the mechanical properties between different CA materials

Understanding the mechanical properties such as elasticity modulus, stress relaxation, and ultimate tensile strength facilitate clinical selection and designing of CAs. Some mainstream and recent emerging CA materials and their mechanical characteristics are listed in Tables 1 and 2.

**Table 1:** The mechanical characteristics for aligners of thermoplastic materials

Aligner	Manufacturer	Material	Elastic modulus(Mpa)	Stress relaxation rate (MPa/min)(2days)	Ultimate tensile strength(MPa)	Author
invisalign	Align Technology, Santa Clara, CA, United State	LD30 (SmartTrack)	842 ± 63	0.0359		Fang [128]
		LD30 (SmartTrack)	61.3 ± 9.18	0.00416 (wet)	40.04 ± 5.00	Shirey [48]
		EX30	103.2 ± 17.3	0.00941 (wet)	64.41 ± 7.25	Shirey [48]
Biolon	Dreve dentamid GmbH. Unna, Germany	PET-G(Polyethyiene terephthalate glycol)	1693 ± 51			Tamburrino [112]
		PET-G	2020			Beldiman [129]
		PET-G		0.0153		Zhang [130]
Duran	Scheu dental. lserlohn, Germany	PET	2366	0.0833		Albertini [131]
		PET	2200			Beldiman [129]
F22	Sweden-Martina, Due Carrare, PD, ltaly	Polyurethane (F22 aligner)	2770	0.1183		Albertini [131]
		Polyurethane (F22 Evoflex)	2104	0.0483		Albertini [131]

**Table 2:** The mechanical characteristics for aligners of 3-D printing materials

Aligner	Manufacturer	Material	Elastic modulus(Mpa)	Stress relaxation rate (MPa/min)(2days)	Ultimate tensile strength(MPa)	Author
Dental LT Clear resin	Formlabs, Somerville, MA, USA	photopolymerizable polymethyl methacrylate	1540 ± 107		40.7 (3days)	Milovanović [130]
TC-85	Graphy, Seoul, Korea	cross-linked with methacrylate functionalization	1186.40			Lee [46]
			42.348		20.7 ± 1.2	Mattle [131]
OD-Clear TF	3DResyns, Barcelona, Spain	Resin (AnyCubic SLA)	38.4 ± 14.7	0.02491	9.34 ± 1.96	Shirey [48]
Material X	Envisiontec, Inc; Dearborn, Mich	Resin (Envision One cDLM)	431.2 ± 16.0	0.04341	28.11 ± 3.75	Shirey [48]

#### Elastic modulus

For thermoplastic materials, CAs are subjected to both short-term and long-term loading forces in the oral cavity. Short-term refers to the period immediately after the aligners are installed on the dental arch, while long-term refers to the process of aligner recovery and force dissipation in the oral environment [43]. In the short-term, thermoplastic materials follow Hooke’s law, exhibiting elastic properties; while in the long-term, thermoplastic materials demonstrate viscoelastic behavior [40]. TPU exhibits elastic behavior under stress and shows greater flexibility as strain increases. It has a strong hysteresis effect which allows notable difference of the stress-strain curves between loading and unloading processes. Furthermore, it displays time dependency which refers to time-dependent increase of strain under constant sustained stress, and cyclic softening which caused progressively softening of the aligners with increasing strain cycling [44]. As a modified version of PET, PETG has become one of the mainstream materials due to its excellent mechanical and optical properties [40]. Below their glass transition temperature, both materials display a definite yield stress. The yield strength and elastic modulus of PETG are lower compared to PET at the same temperature setting. In environments above the glass transition temperature, the stress-strain curves of both materials exhibit a continuous increase in stress as strain increases [45]. The 3-D printing materials have advantages over thermoplastic aligners in eliminating geometric inaccuracies during the thermoforming process and misfit between the aligners and the dental arch caused by shrinkage and expansion [46, 47]. Compared to PETG, the 3-D printing material TC-85 had lower levels of yield stress, elastic modulus, fracture toughness and elastic range [46]. Nic Shirey et al. compared the mechanical properties of two thermoformable materials LD30 and EX30 (LD30 being a single-layer polyurethane, and EX30 a multilayer thermoplastic polyurethane/copolyester blend, both developed by Invisalign) with two 3-D printing materials Material X and OD-Clear TF. They concluded that Material X exhibits a significantly higher elastic modulus under all conditions compared to the other three materials, while the elastic modulus and mechanical strength of OD-Clear TF are significantly lower than the other three materials [48].

#### Stress relaxation

CA is typically made of viscoelastic polymer materials rather than superelastic materials like nickel-titanium alloy springs. It undergoes creep and stress relaxation over time, gradually losing elasticity [49]. Understanding the stress relaxation characteristics of invisible aligners is crucial for optimizing material selection and treatment strategies. Luca Lombardo et al. compared the stress relaxation between polyurethane, PETG, and two multilayer materials PETG/TPU and TPU/PC, showing that polyurethane and PETG exhibited highest values for both absolute stress and stress decay speed, while multilayer materials had more stable and consistent decay rates [38]. In comparison between PETG and TC-85, it was demonstrated that PETG is a non-crosslinked polymer featuring aromatic rings, which facilitates significant π-π stacking interactions between the polymer chains, while TC-85 exhibits a crosslinked network through bifunctional oligomers. The lower molecular weight of TC-85’s monomers results in weaker intermolecular interactions compared to PETG. Consequently, under stress, the molecular chains in TC-85 are more prone to sliding and reorganization, thereby exhibiting more pronounced viscoelastic behavior. As a result, TC-85 undergoes greater stress relaxation over short periods at 37 °C. Nonetheless, its crosslinked architecture allows TC-85 to maintain stable static forces and strain recovery even at elevated temperatures [46]. In a comparative analysis of stress relaxation in four types of orthodontic appliances made from PETG, despite differences in the Ri index (the ratio of hydrogen-bonded carboxyl groups to free carboxyl groups), representing the intermolecular bonding strength), no significant differences were observed in the extent of relaxation [50]. Material X exhibited faster stress decay compared to the three materials LD30, EX30, and OD-Clear TF [48].

#### Ultimate tensile strength

Study on the ultimate tensile strength (UTS) of CA materials is crucial to ensuring that the orthodontic appliance can withstand the masticatory forces. It was found that cured 3-D printing material could withstand higher maximum loads than uncured 3-D materials and thermoplastic materials, while the latter two had similar characteristics by showing plastic deformation [47]. This implies that cured 3-D printing materials may offer higher durability and stability. The ultimate tensile strength of OD-Clear TF is significantly lower than that of EX30, LD30, and Material X [48]. Keller compared the ultimate tensile strengths (UTS) of four different thermoplastic materials (Essix ACE, Taglus, Zendura, Zendura FLX, the first two being PETG and the latter two being polyurethane). It was found that the UTS of Zendura FLX is significantly lower than the other three materials [51].

### The material-dependent mechanical characteristics and clinical performance of CA

A thorough understanding of material characteristics is crucial for guiding clinical practice. The clinical performance of an aligner is to a large extent dictated by the mechanical characteristics of the material. Ensuring continuous and stable orthodontic force during tooth movement is essential in orthodontic treatment [52]. A modulus too low may fail to move teeth effectively, while excessive rigidity may impair the adaptability of aligners and thus compromise the therapeutic results [40]. During the thermoforming process, the elastic modulus of thermoplastic invisible aligners experiences a gradual reduction, which becomes markedly pronounced with an increasing number of cycles [53–55]. Research conducted by Golkhani et al. demonstrated that the elastic modulus of PETG aligners—such as Essix+, Duran Plus, and Essix ACE—exhibited a significant decline after the thermoforming process. In contrast, the elastic modulus of Zendura (polyurethane) aligners remained relatively stable [55]. These findings highlight a crucial consideration in clinical practice: to ensure the delivery of suitable forces, one should not only ascertain the specific type of aligner material but also take into account the variations in its elastic modulus over manufacturing processes.

Unlike the superelastic copper-nickel-titanium, one of the most commonly used materials of archwires, can provide basically constant force within a wide range of deflections [38, 56], CAs are mostly made of polymeric materials, which inherently have characteristics such as stress relaxation and creep. These characteristics lead to time-dependent and strain-dependent changes in the forces applied by CA [32, 35, 36, 56], and make CA fail to provide sufficient force to control the movement trend of teeth as it initially did, causing a series of side effects like the roller-coaster effect [57]. Stress relaxation causes a decrease in the force exerted by orthodontic appliances placed on teeth [41], with force decay exhibiting exponential growth [36]. It was shown that multilayer materials exhibited lower stress relaxation rates and lower initial stress values compared to single-layer materials [38]. A lower stress relaxation rate indicates greater stability of the aligner, allowing for the application of a stable and gentle force during treatment. Additionally, a lower initial stress value means that discomfort for the patient can be effectively reduced when the aligner is first placed in the mouth, enhancing comfort. This underscores the importance for clinicians to possess a nuanced understanding of material properties, enabling the design of meticulously optimized treatment protocols. The intricate mechanical environment within the oral cavity warrants examinations of UTS, Ultimate Compressive Strength, and Ultimate Shear Strength in assessing the holistic performance of CAs. Particularly during the installation and removal, selecting appropriate UTS values is instrumental in ensuring the aligners’ effectiveness and safety throughout treatment. Moreover, a comprehensive understanding of UTS aids aligner designers in refining their thickness and shape, thereby achieving optimal treatment efficacy and enhancing patient comfort. Due to the material characteristics of CA, we can hardly get 100% achievement of the amount of tooth movement as presetted. The discrepancy between predicted and treatment outcomes was around 50% or more [32]. Compensating for this drawback are strategies like added stages of treatment, midcourse correction or overtreatment in presetting [58].

### The geometric characteristics-dependent force transmission of the CA system

CA applies force to the crown by rebound deformation of the aligners. The force is transmitted directly on the crown or through aligner-attachment contact [32]. Both of the two ways take function by effectively “pushing” on the tooth or attachment surfaces, and the effective “pushing” is premised on sufficient “grip” of the tooth, where friction plays a vital role.

#### Application of force by the shape-molding effect

Unlike the forces applied by archwires, the force application by CA is primarily via rebound deformation of the polymeric shells fit over tooth crowns. The pushing force generated by the rebound deformation is fundamentally based on the geometric features of CA, which determine its ability to tightly engage on the occlusal, labial, and palatal surface of the crown, allowing exerting of pushing forces on these surfaces [32, 58]. The direction and acting point of the resultant force in relation to the center of resistance (CR) determines the type of tooth movement. However, the specific points of force application can hardly be determined because of mismatch between aligner and tooth geometries and slipping motions between contact points [32]. The geometric characteristics make it easier for CA to induce tooth intrusion and tipping without the use of attachments [53, 59, 60], while it is not as easily achievable to induce parallel translation, rotation, or torque with this configuration [60–62].

The thickness of CA is another geometric metric that affects force generation. The prevailing view is that within a certain range, the thicker the aligner is, the greater the force would be, and the better control it could exert on tooth movements. Whereas, thickness exceeding a threshold would lead to increased side effects [61, 63]. Other studies suggested that thinner aligners were more suitable for generating forces conducive to tipping, while translation or root movement required thicker aligners [64]. Anyhow, for force generation, one should not merely rely on thickness but rather on combined consideration of thickness and the materials per se.

Research has demonstrated the influences of various trimming line designs on the biomechanics and clinical efficacy of clear aligners. Elshazly et al. employed pressure-sensitive film to meticulously analyze the force distribution exerted on individual teeth across different trimming line configurations. Their findings revealed that, under identical conditions, straight trimming lines generated greater levels of both active and passive pressures in comparison to scalloped designs [65]. Moreover, extended trimming lines further intensified the pressure on the teeth [65]. Subsequently, Elshazly et al. conducted a finite element analysis to investigate the force distribution throughout the entire dental arch. They found that the resultant force associated with straight trimming designs surpassed that of scalloped configurations, with the overall force increasing in correlation with the extension of the aligner edges [66]. The normal contact force exhibited an uneven distribution across the surface, predominantly concentrated on six key areas: incisors, mesio-incisal, disto-incisal, middle, mesio-cervical, and disto-cervical [66].

#### Application of force in conjunction with auxiliaries 

The incorporation of attachments into the CA system enriched the contact relations between aligners and the surfaces being pushed. The geometric features of CA were thus altered, making it possible to achieve movements that are unachievable with CA alone [32, 42, 55, 60, 67]. The attachments were categorized into two types: conventional and optimized. Conventional attachments, with rectangular, beveled, ellipsoidal, or semi-spherical shapes and predefined dimensions, are commonly placed on any tooth, and can be oriented in various directions. Optimized attachments, with shapes, dimensions, and positions tailored to tooth anatomy and desired movement, are positioned by technicians. The position, dimension, or orientation cannot be altered in Clincheck. Originally designed to create specific force systems for controlling canine and premolar rotation, optimized attachments offered supplementary and sometimes more precise approaches to treatment [33]. Optimized and horizontally oriented rectangular attachments provided superior mesiodistal control compared to vertically oriented rectangular attachments [68, 69]. Gao et al. found no difference between conventional horizontal and vertical rectangular attachments in strain-stress and tooth displacement trends during molar distalization in a finite element analysis [70]. Horizontal rectangular attachments yielded higher stress at the root apex and greater amount of extrusion of maxillary central incisors, compared to rectangular beveled and ellipsoid attachments [71].

Although it was demonstrated (Jedliński et al.) that using attachments yielded better treatment outcomes compared to treatment without attachments, attachments do not guarantee better movements [72]. Mismatch happened between the attachments and the aligner would cause unpredictable or unwanted tooth movements. Moreover, whether attachments are present or not, it is impossible to prevent lingual tipping of the anterior teeth [73]. Alterations on shape and position of the attachments were tried to derive different biomechanical design that aims to improve treatment outcome. Attachments adhered to the palatal surface of the anterior teeth were found to derive better tooth movements than those adhered to the labial side [72]. Hong et al. reported an overhanging attachment that generated less stress in the periodontal ligament (PDL) compared to conventional attachments. The decreased distance between CR and the overhanging attachment reduced the generation of moments, minimizing activation of unintended forces beyond the preset [74].

The implementation of temporary anchorage devices (TADs) enables enhancement of anchorage control in the CA system [75–78]. Jia et al. analyzed the force in microimplant-assisted molar distalization using a 3-D finite element method. They demonstrated that the Class II elastic traction from maxillary micro-implants to the precision cuttings on aligners had better efficacy than that directed towards bonded buttons on teeth, in mitigating anchorage loss during en-mass distalization of the molars [75]. A recent study demonstrated that in the TADs-incorporated clear aligner system, the effective contribution ratios of molar distalization and anchorage reinforcement significantly increased with the application of greater force magnitudes. However, this escalation in force also led to adverse movements, such as molar tipping, rotation, and an increase in stress on the molars. Clinically, the optimal force recommended for this procedure is approximately 4.5 ounces, or roughly 130 g [76]. Several studies have indicated that utilization of TADs for precise control of anterior tooth intrusion often necessitates the application of supplementary force and torque to attain the intended movement [77]. Moreover, by meticulously manipulating the line of action of the force or strategically repositioning the TADs, clinicians can effectively regulate the degree of labiolingual tipping, thereby enhancing treatment outcomes [78].

Precision cut design is another geometric feature that influences the force system generated by CAs [75]. One of the most commonly encountered examples is management of Class II malocclusions where precision cuts are employed on maxillary canine and mandibular molar areas to facilitate mandibular mesialization using Class II elastics or TADs-aided maxillary distalization [79]. Precision cut hooks in the aligners deliver more evenly distributed force on teeth than application of button cuts and orthodontic buttons on individual tooth. The latter tends to have higher risk of undesired tooth rotation and subsequently disengagement of aligners. Nonetheless, challenges arise when the clinical crowns are short or when the precision cut hooks are subjected to substantial forces, potentially leading to the detachment of clear aligners from the dental surfaces, which results in inappropriate tooth movement [79]. In a finite element study, button-binding on the canines was reported to be more effective than precision cut hooks for distal movement of the maxillary dentition [80].

#### Friction between the contact surfaces

Friction plays a pivotal role in the efficacy of force transmission in the aligner systems. The dynamic between the aligners and attachments or enamel surfaces significantly influences treatment outcomes. A relative motion between the interface can lead to the dissipation of mechanical energy intended for tooth movement, thus diminishing treatment efficacy. Conversely, excessive adherence between the aligner and the teeth could hinder the removal process of the aligner, complicating patient compliance and overall treatment experience [81]. The natural tooth morphology influences the efficacy of CA treatment, by affecting the friction between tooth-aligner contact surfaces. The heterogeneity of oral anatomy among individuals means that each patient presents unique points of contact, resulting in varying frictional interactions. Takara highlighted that the extent to which the aligner margin envelops the teeth directly correlates with increased frictional grip [81]. Furthermore, the geometrical characteristics of attachments, such as their size, shape, and placement, play significant roles in modulating this grip [81, 83]. The coefficient of friction is not uniform across different regions of the dental arch, which presents challenges in modeling and simulation [82]. Standard simulations typically assume a friction coefficient of 0.2 [84]; however, adjusting this value to reflect more specific clinical scenarios could enhance the predictive accuracy of treatment outcomes. Additionally, the wear interactions among the upper and lower aligners, and between aligners and both teeth and attachments [86, 87], must be accounted for. This wear not only potentially alters the friction coefficient over time but also raises concerns regarding the ingestion of microplastic particles (MP) [85], which could have health implications. Studies show that different wear protocol affect the accuracy of treatment [106] but current research is scant on how wear affects the mechanical properties, such as friction, of clear aligners — a gap that needs to be addressed to fully understand the biomechanical behavior of CAs.

## Methods for studying the biomechanics of CA

### Finite element analysis (FEA)

The FEA was the most commonly used method for studies of biomechanics in orthodontics. It enables simulation of physical properties of dental and periodontal structures periodontal ligaments, measurement of stress and visualization of actual displacements. It has advantages including non-invasiveness, repeatability in controlled experiments [57, 88], and compatibility with multi-physical field coupling and optimization design.

#### Material parameters

Below we summarized the material parameters used in biomechanical studies of CA (Table 3), hoping to provide reference for enhancing the comparability of research results and improving the accuracy and reliability of finite element models.

**Table 3:** the material parameters used in biomechanical studies of CA

Material	Elastic Modulus(MPa)	Poisson’s Ratio	author
Enamel	84,100	0.33	Hemanth (2015) [20]
Dentin	14,700	0.31	Liao (2016) [90]
Tooth (not distinguishing between Enamel and Dentin)	19,600	0.30	Fan (2022) [16] Cheng (2022) [21] Kang (2023) [22] Guo (2023) [25] Liu (2022) [26] Lyu (2023) [27] Zhu (2023) [49] Alhasyimi (2024) [61] Wang (2023) [93] Cheng (2022) [96]
	20,000	0.30	Cattaneo (2008) [89] Zhou (2019) [94]
	17,000	0.30	Gandhi (2021) [98]
	18,600	0.31	Xia (2024) [74]
	20,300	0.26	Geramy (2024) [17]
	200,000	0.30	Guo (2023) [25]
Cancellous bone	1370	0.30	Guo (2023) [25] Cattaneo (2008) [89] Xia (2024) [74]
	13,400	0.38	Geramy (2024) [17]
	13,700	0.3	Hemanth (2015) [20] Guo (2023) [22] Xia (2024) [74]
Cortical bone	13,700	0.3	Hong (2021) [75]
	14,700	0.31	Liao (2016) [90]
	34,500	0.3	Hemanth (2015) [20]
periodontal ligament (not include bilinear and hyperelastic)	0.69	0.45	Hemanth (2015) [20] Zhu (2023) [49] Wang (2023) [93] Cheng (2022) [96]
	50	0.45	Cattaneo (2008) [89]
	0.67	0.45	Liu (2022) [26]
Clear aligner	528	0.36	Fan (2022) [16] Zhu (2023) [49] Liu (2022) [44] Alhasyimi (2024) [61] Gao (2023) [60] Wang (2023) [93]
	816	0.36	Cheng (2022) [21]

In reality, the collagen fibers within the PDL are non-directional, exhibiting nonlinear behavior. Some studies employed hyper-elastic models to simulate the Young’s modulus of the periodontal ligament (PDL) [89], and to obtain the stress-strain curve [90]. Although some studies showed no significant difference between the linear and nonlinear stress-strain model of the PDL in simulating tooth movement [91], the model may have influence on determination of the critical stress ratio [92]. A bilinear model offered operational simplicity while ensuring a certain level of accuracy [90]. Typically, the thickness of the PDL is about 100 micrometers, and the simulation of the alveolar socket can be achieved through Boolean operations [67].

The parameter settings of the alveolar bone were different between studies, with some of the studies using the elastic modulus of cancellous bone [21, 67, 92], and the others using parameters of cortical bone [16, 22]. These differences in settings may have impact on the simulation results, whereas the impact is much smaller than the parameter settings of the teeth and PDL. It is difficult to precisely simulate the real structures of the alveolar bone. It is worth noting that despite the consistency of parameter settings between some studies [16, 25, 49, 67, 92], experimental tests might be needed before performing finite element analysis to obtain more accurate parameters.

#### Simulating orthodontic forces and obtaining of the optimal force

In the field of CA, there are mainly two types of problems being addressed through finite element analysis. The first is how to achieve an accurate simulation given certain presets. The second is to determine the optimal orthodontic forces for desired movements. The essence of the first problem is how to accurately simulate orthodontic force and apply the force in the software.

The orthodontic forces in fixed appliances were simulated by calculating the rebound force after bending of the archwire [93], or by calculating the stress inside the bracket [94]. The former method was analogous to the currently mainstream approach for simulating orthodontic forces in CA. Displacement loads were applied to the aligners, and the resulting force upon returning to the original position was calculated as the orthodontic force [9, 95]. We speculated that the orthodontic force in the CA system might also be measured as the stress on the inner surfaces of the aligners, or as the stress on the surfaces of the attachments and tooth crowns. This method measures the force more straightforward but may face difficulties caused by the variable and sometimes untight contact relations between crown or attachment surfaces and inner surfaces of aligners. Calculations of the resultant force might also be complicated by the complex morphologies of the crowns.

For obtaining the optimal orthodontic force, the premise with paramount importance is to obtain an accurate location of the center of resistance (CR). Determination of the CR location precedes the analyses of force systems and tooth movement. FEA has been widely used to determine the location of CR, though with limitations. Some of the studies determined the CR locations in a two-dimensional plane [96], while others proposed CR determination in a 3-D space [97]. We summarized the method as below. After modeling the individual teeth and establishing the standard planes, forces and torques were applied at randomly selected points in each plane. The CR location was identified as the point where the moment equaled zero. This method applies similarly to multi-tooth systems. In practice, it is difficult to locate the precise position of CR, as its location is affected by multiple factors including root length and volume, root shape, bone density, thickness and physical properties of the PDL, all being factors with great inter-individual variability [90].

Another method to obtain the optimal force was based on the volume average hydraulic pressure of the PDL: quantifying VAHS (Volume Average Hydraulic Pressure). Continuous orthodontic force is applied externally, with each force corresponding to a VAHS value. The range for the optimal force was determined when the VAHS fell into the range from 4.7 kPa to 16 kPa [89].

### Other methods for studying the biomechanics of CA

In 1992, Planert et al. established a pioneering three-dimensional torque measurement apparatus [82]. Concurrently, the “Orthodontic Measurement and Simulation System” (OMSS) gave rise to the Robot Measurement System (RMS), a significant advancement in the field [98]. This innovative system leverages computer-assisted diagnostics to meticulously analyze the dynamics of tooth movement alongside the exerted forces and torques, facilitating the simulation of complex dynamic movement sequences through intricate programming that correlates torque values with robotic actions [99]. In 2009, an orthodontic simulator known as OSIM was developed by Badawi et al., incorporating Nano17 sensors and an aluminum dental model. This simulator is capable of concurrently measuring the forces applied by orthodontic devices across the entire dental arch. Each tooth is individually linked to the system’s sensors through both vertical and horizontal connectors, while rotary connectors enable three-dimensional movement of the teeth with a precision of 0.01 millimeters, accurately simulating various types and severities of malocclusions [100]. Nano17 sensors, noted for their compact size and exceptional precision, have become the sensor of choice in the realm of invisible orthodontic appliances [101, 102]. In 2021, Kaur et al. employed this force measurement system to assess the forces and torques exerted by 0.75 mm clear aligners on three distinct teeth in the upper jaw and their adjacent counterparts [103]. By 2023, Hu et al. had adopted Nano17 mechanical sensors to construct a three-dimensional mechanical model aimed at analyzing the forces and torques involved in the distal movement of various groups of molars [100]. In addition to the widespread use of the Nano17 six-axis sensor, Midorikawa created The Fourteen Orthodontic Sensing Device (FORSED), which utilizes the CFS018CA201 six-axis force/torque sensor coupled with 3-D printed resin dental models to measure orthodontic forces and torques [50]. In 2018, Mencattelli and colleagues introduced a device that could simultaneously measure the three-axis forces and torques on fourteen mandibular teeth [81]. Despite the lower adaptability of biomechanical test benches compared to computational models, they provide a robust means for examining the intrinsic physical characteristics of materials, specimens, and instruments without the influence of subjective parameters [99].

The photoelastic methods were constructed based on the principle of photoelasticity that the refractive index of transparent materials changed under different stresses. Polarized light passed through the tested material, and the stress in the material altered the polarization state of light, which could be observed through polarizing mirrors. By analyzing these optical patterns, it’s possible to qualitatively and quantitatively described the stress distribution within the material [104].

Another method was measuring the surface strain of materials using strain gauge technique. The strain gauge was attached to the material surface and underwent expands or contracts when the material was subjected to force. The deformation of the gauge caused changes in its resistance, which was measured and calculatively converted to the surface strain of the material [105]. Moreover, some researchers analyzed teeth movement by comparing the digital models between virtual teeth alignment and actual treatment outcome. The digital models of virtual alignment and intraoral scanning were superimposed or compared within the same 3-D software [106].

## Biomechanical analyses for CA-induced tooth movement

### Intrusion

To some extent, the intrusion that CA aims to achieve isn’t solely vertical movement but rather a displacement with a backward or downward direction accompanied by tipping [32]. Under the same presets, the canines bear the greatest force in the vertical direction irrespective of individual intrusion or combined intrusion of the anterior teeth [9]. Individual intrusion of anterior teeth resulted in the greatest extrusive forces on the premolars [9]. In a FEA model of full maxillary arch intrusion using CA and temporary anchorage devices (TADs), with intrusive forces applied from attachments between the canines and first premolars and between the first and second molars, greater intrusion was recorded in the posterior segment than the anterior teeth, undesirable movements occurred such as buccal tipping of canines, distal inclination of incisors and mesial rotation of molars [17]. During molar intrusion, an uncontrollable counterclockwise moment would be generated irrespective of the attachment positions, resulting in mesial tipping of the second molars [17].

### En-masse retraction of anterior teeth

When activating presets for retraction only, the maxillary central incisors experienced the largest lingually directed force, the maxillary second molars experienced the second largest lingually directed force, the canines experienced the greatest distal force, and the first and second molars experienced a significant mesial force [12]. In the vertical dimension, the incisors experienced extrusive force, while the canines and second premolars experienced intrusive forces [15]. These force distributions provided explanations for the observed tooth movements including incisor retraction with lingual tipping and extrusion, distal tipping and extrusion of canines, and mesial tipping and intrusion of second premolars [15, 59]. Using a FEA model, Yang et al. revealed that CA exerted a protraction force on the maxillary first molars during space closure after premolar extractions. The protraction force comprised a mesial force in the sagittal plane and a lingual force in the buccolingual direction, leading to undesirable mesial and lingual tipping of the molars. Employment of anchorage-prepared appliances counteracted this trend [29].

During en-masse retraction, the stress on the roots was mainly concentrated at the labial cervical region and palatal non-apical region for the maxillary central incisors, the labial apical and cervical regions for the canines, and the mesial cervical region and apical areas for the second premolars [18, 59]. For the PDL, the maximum stress was located at the root apex of the canines, followed by the cervical region of the canines and central incisors. The mesial cervical area of the second premolars and the distal cervical area of the second molars also exhibited high stress levels [15, 92]. For the alveolar bone, the stress was primarily concentrated in areas between the canines and second premolars [67]. Using a FEA model, Liu et al. preseted overtreatment by rotating the anterior part of the appliance counterclockwise around the center of the first premolar aligner vacuoles, and found that the overtreatment resulted in well-distributed stress along the whole arch [67]. In contrast, in the model without overtreatment, the stress was concentrated on the cervical and apical regions on the labial side and the non-apical regions on the palatal side of the anterior segment. These results indicated overtreatment as an approach to facilitate bodily retraction of the anterior segment and attenuate orthodontic force-induced root resorption.

### Intrusion combined with en-masse retraction

In addition to activating retraction, a certain amount of intrusion was applied to address the side effect during space closure, as CA lack rigidity and were prone to inducing torque loss and undesired extrusion of the anterior teeth [19]. In the labial-lingual direction, the central incisors experienced the greatest lingually directed force, while the force was labially directed for canines and lingually directed for second premolars. Compared to solely retraction, activating the preset of intrusion combined with en-masse retraction led to greater force on the incisors and canines in the labial-lingual direction, decreased distal force on canines and increased mesial force on premolars, and changed direction of force that extruded the second premolars [15]. The preset of intrusion combined with en-masse retraction yielded more evenly distributed stress in the PDL, and induced less retraction and more intrusion of the anterior teeth.

### Torque

**Fig. 2:**
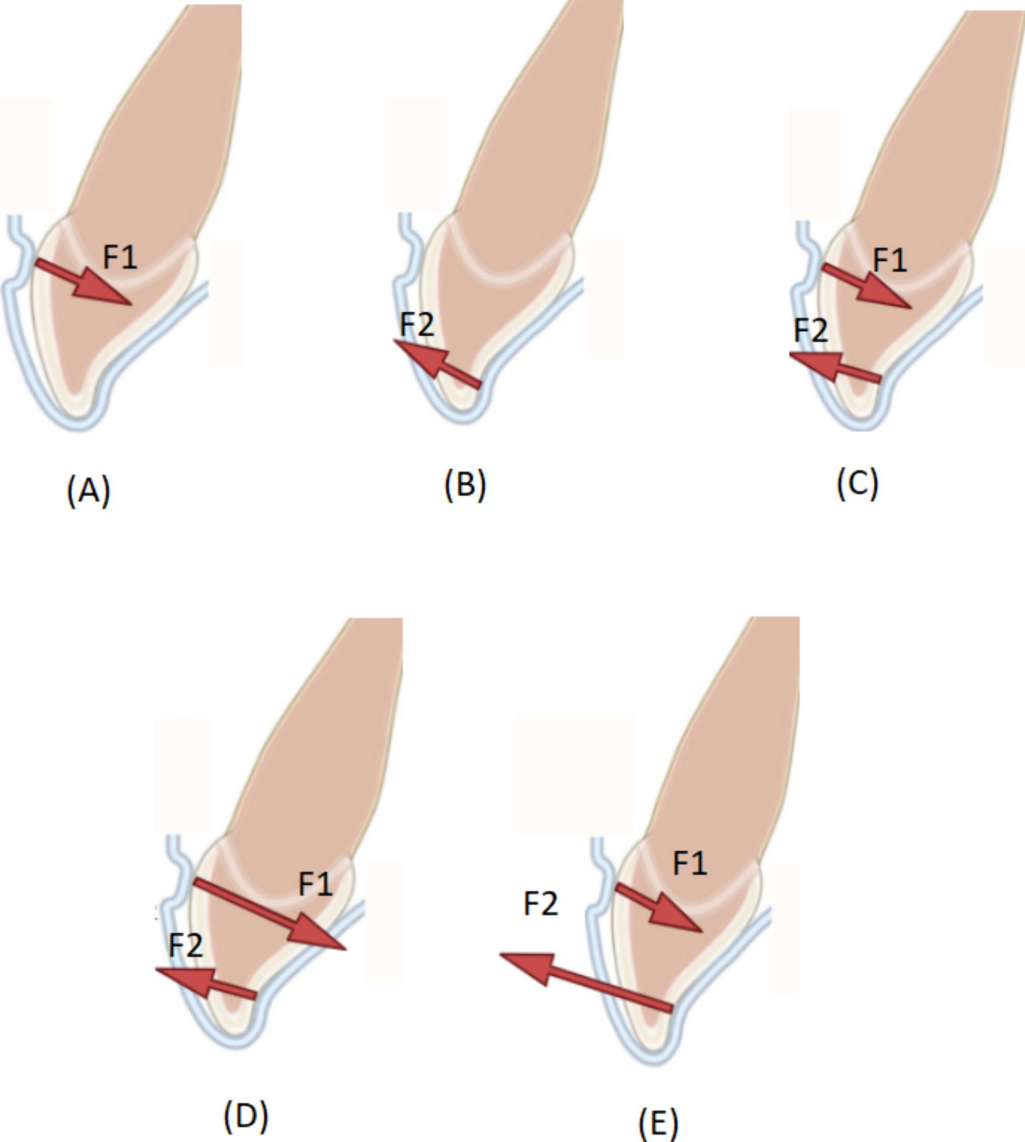
Generation of a couple in the aligners by using power ridges **(A)**. The geometric deformation of the aligner applies force (F1) to the crown on the contact point of the power ridge. This force induces lingual tipping of the tooth. **(B)**. An additional force (F2) is generated at the lingual side when the lingually tipped crown presses on the inner surface of the aligner. **(C)**. F1 and F2 constitute a couple when F1 equals F2 **(C)**, or a pair of forces that could be decomposed into a couple and an additional force when F1 is larger **(D)** or smaller **(E)** than F2

To induce torque movement, a couple is needed [107]. A Couple is a pair of forces of equal magnitude acting in parallel but opposite directions. The geometric characteristics of CA and the contact relations between aligners and tooth crowns determine that we can hardly obtain a pure couple merely by an aligner unless incorporating the usage of auxiliary elements like attachments or power ridges. In most cases, the resultant forces generated by deformation of CA can be split into two or multiple components, some of which may cause uncontrollable tooth movements such as intrusion, lingual inclination, and extrusion [22]. In general sense, CAs are not good at torque movement. However, the force system can be changed by altered geometric features of the CA system, in which designs of attachments and power ridges are of current interest. The generation of a couple by using power ridges in aligners is illustrated in Fig. 2. Different geometric contact relationships were reported to have various effects on torque movement [12, 22, 28]. Under the same activation settings, the torque values produced by using a power ridge are greater than using a horizontal ellipsoid attachment. Application of power ridge also caused reduced vertical component of force [12]. Moreover, as the height of the power ridge increased, the distance from center of rotation to the CR decreased, and the labiolingually rotational angle of the maxillary central incisors decreased, leading to a greater tendency for translation [22]. In addition, the length of the power arm also affects torque. Increasing the length of the power arm results in the resultant force being closer to the center of resistance, causing less torque movement compared to having no power arm [28]. Further studies and clinical practice are warranted to attempt new approaches for better control of torque movement.

For teeth undergo torque movement, the stress distribution varied dependent on numerous factors including the inclination of the teeth, the shape of the aligner, the shape and type of attachments, the position and design of auxiliary elements like power ridge and power arm, etc [11]. Stresses were primarily concentrated on the cervix and root for incisors and canines, and on the cervical region for second premolars. The peak values of stress in the PDL of the canines were smaller than those of the second premolars. As the degree of inclination of anterior teeth increased, the peak stress in the PDL tended to decrease, except the central incisors [22]. Moreover, the peak stress values were affected by the position of the power ridge. As the height of the power ridge increased, the peak stress on the tooth root increased, though with no significant change in the stress in the alveolar bone or PDL [22].

### Rotation

Using CAs of the same material and thickness, for the same predetermined rotation angle, the moments required for mesial rotation of incisors and canines were smaller than those required for distal rotation [23, 24], suggesting that in clinical practice mesial rotation might be easier to achieve than distal rotation. Additionally, a thicker CA influenced the rotation of teeth by providing increased magnitude of force [23], potentially resulting in greater pressure on PDL and and increased risk of root resorption. Materials and attachments also affected the orthodontic forces involved in tooth rotation [9, 24]. In the same predetermined conditions without attachments, the torque obtained from the geometric deformation of the CA will significantly decrease. Practically, without attachments, the rotation movements attempted to be achieved by CA were frequently accompanied by undesirable movements, of which the most frequently reported was intrusion [24]. Usage of attachments led to increased moment exerted on the teeth and less occurrence of intrusion [9].

### Molar distalization

During molar distalization, the tensile and compressive forces on the roots of the molars are significantly higher than those on the anterior teeth, with the central incisors bearing the lowest stresses [25]. The highest compressive stress was concentrated in the labial cervical and apical regions of the PDL for the maxillary anterior teeth, and the highest tensile stress was distributed in the lingual cervical region. For maxillary canines, the highest compressive stress was concentrated in the mesial-buccal cervical and apical regions. The stress on the PDL of posterior teeth was significantly greater than that on anterior teeth. The stress on the alveolar bone was mainly distributed on the labial surface, concentrating in the cervical and apical regions [26]. The lingual and distal regions of the premolars were also stress-concentrated areas. Usage of TADs and elastics reduced stress in the PDL and yielded a more evenly distribution of stress [25, 26].

### Molar mesialization

molar mesialization, especially the long-distance mesialization, is one of the most difficult task for CA-induced movements. Very limited evidence was found for its biomechanical analysis clinically or experimentally. At the initial stage of mesialization, compressive stresses were mainly observed at the mesial root cervix and distal root apex of molars, while tensile stresses were concentrated at the distal root cervix and mesial apex [27]. Lyu et al. utilized iterative finite element analysis to simulate long-term mesial movement of molars. They found that the stress on the roots decreased after an initial loading period, with high compressive stress concentrated at the buccal cusps and cervical areas, and tensile stress at the distal root cervix. Towards the end of the treatment process, compressive stress was observed on the mesial side, while tensile stress was on the distal side. The changes in stress distribution on the roots and PDL shed light on the unforeseen tooth movements that may occur during the mid-treatment phase, such as lingual rotation and intrusion of the molars [27]. Understanding of this biomechanical basis help clinicians anticipate the undesirable movements and optimize treatment strategies.

### Collective movement of multiple teeth within an arch

Beyond segamental analysis of tooth movements of specific types or of specific segment of the arch, grasping the intricate biomechanical dynamics of movement and foundational principles governing the collective movement of multiple teeth within an arch is crucial for informed clinical decision-making. The mechanical frameworks governing the movement of multiple teeth were categorized into synergistic mechanics and antagonistic movements. Representative examples of synergistic mechanics include combinations such as expansion paired with hinge rotation, and posterior compression coupled with anterior protrusion. Antagonistic movements may include posterior expansion and anterior protrusion, rotation and extrusion, etc [108]. In antagonistic mechanical systems, the orthodontic forces deriving therapeutic outcomes may also introduce additional uncontrollable side effects. Rather than merely raising the force, incorporating in the system additional designing such as TADs or attachments would be more efficient solution to optimize treatment outcomes [108].

Another case worthy of note is when TADs were used. Liu et al. investigated the biomechanical effects of direct and indirect strong anchorage with TADs. With direct strong anchorage, the retraction force caused intrusion of the mid-arch, extrusion of the anterior teeth and arch ends [109]. The direct strong anchorage resulted in relieved incisor extrusion compared to the indirect strong anchorage system, attributing to the vertical component of the elastic retraction. The indirect strong anchorage system provided better prevention of mesialization of molars but may aggravate tipping of the posterior teeth due to premolar intrusion [109]. 

## Discussion

The orthodontic force generated and transmitted by CA is material-dependent and geometric-dependent, two aspects by which the mechanical characteristics could be understood. Currently, the mainstream materials for CA are thermoplastic materials, which have mature manufacturing technique with good biocompatibility [91]. However, the thermoplastic aligners have limitations that the materials were influenced by thermoforming processes [53], and environmental factors in oral cavity, leading to uncontrollable and unpredictable changes in mechanical properties like elastic modulus and yield strength [110]. The emerging 3-D printing materials have advantages in resolving some of the above limitations such as geometric inaccuracy or misfit resulting from thermoforming processes [47, 67, 111]. However, for the currently tested 3-D printed aligners, there is thusfar no evidence to support improvements on the intrinsic mechanical behavior of the material. The current dominant 3-D printing technologies encompass fused filament fabrication, selective laser sintering, selective laser melting, and Stereo Lithography Appearance. The Digital Light Processing (DLP) technology, renowned for its precision and expedited printing capabilities [112], stands as a preeminent method for 3-D printing in orthodontics, and was anticipated to sustain its dominance in the field in the near future [113]. The application of 3-D printing materials in CA are currently largely in the experimental stage, with a notable absence of commercially viable products [114]. Challenges are still faced in resolving inaccuracies in printing orientation and deviations in thickness from design specifications [114]. More tests and examinations are warranted to improve the manufacturing processes and explore the clinical applicability and efficiency.

The force transmission of the CA system is determined by geometric features which dictate the contact relations between the aligner and the surfaces being pressured [115]. Alterations on the aligner morphologies per se and involvement of various auxiliary elements diversify and enrich the geometric features to a large extent, allowing development of biomechanically optimized patterns of force transmission [33, 115]. Thus far it is far more difficult to move the root than to move the tooth crown. CA is facing challenges to improve the efficacy in achieving long-distance translation, rotation, and root torquing [58, 60, 63], all tooth movements that would greatly enlarge the scope of CA. These challenges are fundamentally biomechanical challenges.

The ever-evolving computer technologies have boosted fast advancement in optimization of aligner designs [39], heralding a paradigm shift in future orthodontic interventions. Artificial intelligence (AI) is applied to improve the aligner designs by focusing on clinical monitoring and outcome prediction [116, 117]. The optimized attachments are one of the examples. Large models are developed to customize the shape and positioning of attachments to improve therapeutic outcomes. However, notable discrepancies were still observed between expected and actual tooth movements [118, 119], in certain cases the efficacy of these technologically enhanced attachments parallels or falls short of conventional or non-attachment strategies [120]. These issues may have roots in the biomechanical aspects. There remains a substantial gap in research concerning the model explainability and the intrinsic biomechanical foundation of AI-aided aligner designs. This niche exhibits profound research significance and untapped market potential. The AI-driven optimization of aligner designs could be expanded to more geometric features such as overtreatment angles and strategies. Leveraging AI to tailor treatment strategies could be expanded to more aspects such as biomechanical calculations and explorations.

FEA is currently the most widely adopted tool for studying the biomechanics of CA. Despite its advantages, FEA has remarkable limitations. Firstly, it is practically unachievable to accurately set the parameters of the position of CR. The settings of the loads may also be challenging because the contact parameters, which include the contact area, pressure distribution, and the position of contact points between the aligner and the teeth, are all highly variable in reality. The digitally-simulated idealized models do not perfectly match the real-world states [30]. The FEA models simulated uniform aligner thickness, while in reality the aligner thickness varies significantly across the entire tooth surface [31]. The questionable accuracy of parameter settings lead to uncertainty of the FEA-based results [121]. Rather than the virtual simulation, explorations of some real-world examinations by approaches like force sensors or converters may make substantial progresses in biomechanical research.

Patient compliance is a pivotal factor influencing the success of clear aligners. Existing literature underscored that, in order to achieve optimal therapeutic outcomes, it is essential for patients to wear their aligners for approximately 20 to 22 h each day [122]. Should the mechanical design of an aligner effectively address the critical criteria of comfort, stability, and the ability to visualize treatment progress, it is likely to substantially enhance patient adherence, thereby facilitating improved clinical results. At present, the majority of researches concerning patient compliance with clear aligners predominantly emphasized the correlations between compliance and various factors, alongside elements of remote monitoring [123–125]. There exists a notable scarcity of literature that rigorously investigates, from a biomechanical perspective, how patient comfort and compliance fluctuate across diverse clinical cases and treatment scenarios. Furthermore, researches exploring the optimization of design and treatment strategies through a biomechanical lens remain insufficient. A comprehensive and nuanced investigation into this domain is critically warranted.

## Conclusions

From the perspective of biomechanics, CAs are currently facing challenges in improving material properties and force transmission. Explorations of novel materials and manufactory techniques are essential to derive revolutionary advancement in the mechanical properties of CA. Modifications of the morphological or geometric design of the CA system could help explorations of biomechanically optimized patterns of force transmission. Rather than virtual evaluation of force, developmemt of real-world force sensing and monitoring system would facilitate and accelerate these exploration processes.

## Data Availability

Not applicable.
